# mRNA Interferase *Bacillus cereus* BC0266 Shows MazF-Like Characteristics Through Structural and Functional Study

**DOI:** 10.3390/toxins12060380

**Published:** 2020-06-08

**Authors:** Sung-Min Kang, Ji Sung Koo, Chang-Min Kim, Do-Hee Kim, Bong-Jin Lee

**Affiliations:** 1 The Research Institute of Pharmaceutical Sciences, College of Pharmacy, Seoul National University, Gwanakgu, Seoul 08826, Korea; men0528@snu.ac.kr (S.-M.K.); koojisung@snu.ac.kr (J.S.K.); kt912@snu.ac.kr (C.-M.K.); 2 College of Pharmacy, Jeju National University, Jeju 63243, Korea; doheekim@jejunu.ac.kr

**Keywords:** toxin–antitoxin system, mazF, type II, toxin, mRNA interferase, X-ray crystallography

## Abstract

Toxin–antitoxin (TA) systems are prevalent in bacteria and are known to regulate cellular growth in response to stress. As various functions related to TA systems have been revealed, the importance of TA systems are rapidly emerging. Here, we present the crystal structure of putative mRNA interferase BC0266 and report it as a type II toxin MazF. The MazF toxin is a ribonuclease activated upon and during stressful conditions, in which it cleaves mRNA in a sequence-specific, ribosome-independent manner. Its prolonged activity causes toxic consequences to the bacteria which, in turn, may lead to bacterial death. In this study, we conducted structural and functional investigations of *Bacillus cereus* MazF and present the first toxin structure in the TA system of *B. cereus*. Specifically, *B. cereus* MazF adopts a PemK-like fold and also has an RNA substrate-recognizing loop, which is clearly observed in the high-resolution structure. Key residues of *B. cereus* MazF involved in the catalytic activity are also proposed, and in vitro assay together with mutational studies affirm the ribonucleic activity and the active sites essential for its cellular toxicity.

## 1. Introduction

Toxin–antitoxin (TA) systems are prevalent in prokaryotes and function to regulate cellular growth in response to stress such as antibiotic exposure, nutrient starvation, heat shock, and DNA damage. Initially, TA systems were discovered as a part of the plasmid maintenance system, in which only the daughter cells harboring the vertically transferred TA operon can survive [1,2,3,4,5]. In addition to the primary maintenance function, TA systems are important for bacterial survival. The TA systems are involved in many cellular processes, including cell growth, cell persistence, cell dormancy, biofilm formation, antibiotic resistance, DNA replication, translation, cell division, cell wall synthesis, and cell apoptosis [6,7,8,9,10,11].

TA systems are a two-component system, composed of a toxin and an antitoxin usually sharing the same operon. Currently, TA systems can be categorized into six phenotypes (I-VI) by the nature of each component and the regulatory mechanisms of the antitoxins to its cognate toxins. In the case of RNA antitoxins, direct inhibition of mRNA encoding toxins and protein toxins constitute the type I and type III systems, respectively. Similarly, protein toxins are directly inhibited by their counterpart protein antitoxins in type II TA system. As for indirect regulatory mechanisms, protein antitoxins counteract the activity of protein toxins in type IV TA system and cleave mRNA encoding protein toxins in type V TA system. Lastly, in type VI TA system, protein toxins are degraded by specific proteases and lose their toxicity as a result of complex formation with protein antitoxins [12,13,14,15,16].

Typically, in type II TA system, the rather stable toxins have much more invariant characteristics than their labile and flexible cognate antitoxins. Upon degradation of antitoxins, free toxins can function in postsegregational cell killing, abortive infection, and even bacterial persistence. Thus, it has been regarded that bacterial persistence would indeed be closely related to type II TA systems due to the cellular process-specific nature of type II toxins [17,18,19,20,21]. One of the best characterized type II toxins is mRNA interferase MazF toxin, belonging to the MazEF family. Comprehensive bioinformatic analyses have shown that MazF is widely distributed in both Gram-positive and Gram-negative bacteria, allowing an in-depth understanding of the consequences of stress and MazF activation on the physiological effects of the bacteria [6,22,23]. In addition, understanding of the cutting specificity of MazF on its target RNA is well established. Specifically, the MazF toxin acts as a ribonuclease and cleaves mRNA in a sequence-specific and ribosome-independent manner, but in some cases also cleaves rRNA at specific sequences, exerting its toxicity [17,24,25].

*Bacillus cereus* is a Gram-positive, facultatively anaerobic, and rod-shaped bacterium that is well known for its association with foodborne illness [26]. Recently, the pathogenicity potential of the bacteria gained attention due to its ability in causing serious and fatal nongastrointestinal infections [27]. As TA systems are involved in several cellular responses such as stress response, bacterial persistence, and biofilm formation, there is a clear linkage between TA systems and the virulence of the bacteria [28]. For this reason, research was conducted on the putative MazF toxin, mRNA interferase BC0266.

Here, we report 2.0 Å X-ray crystal structure of mRNA interferase MazF as the first toxin structure in the TA system of *B. cereus*. Similar to that of other MazF toxins, *B. cereus* MazF adopts a PemK-like fold and also has an elongated loop between the β1 and β2 strands. Further sequence alignment with several other MazFs supported the key residues in ribonucleic catalysis. Finally, in vitro ribonuclease activity test together with site-directed mutational studies demonstrated that *B. cereus* MazF exhibits a clear ribonuclease activity and its key residues play a significant role in exerting its role as a ribonuclease. In conclusion, we show that mRNA interferase BC0266 belongs to the MazEF family and is the first reported MazF toxin structure in the TA system of *B. cereus*. This is an important study to investigate the diversity of the TA system among many different bacterial strains.

## 2. Results and Discussion

### 2.1. B. cereus MazF Adopts a PemK-Like Fold

Crystals of *B. cereus* MazF were diffracted to high resolution (2.0 Ǻ) and the final structure was determined using the molecular replacement method with the MazF structure from *Mycobacterium tuberculosis* (PDB code 5XE2) [29]. The structure was refined to *R_work_* factor 18.9% and *R_free_* factor 22.5%. All 116 amino acids were present and lie within the Ramachandran favored region. Crystals had *P3_1_21* space group with the unit cell dimensions of *a* = *b* = 60.648 Å, *c* = 76.247 Å, and α = β = 90.0°, γ = 120°. Detailed crystallographic data collection and refinement statistics are summarized in Table 1.

**Figure 1:**
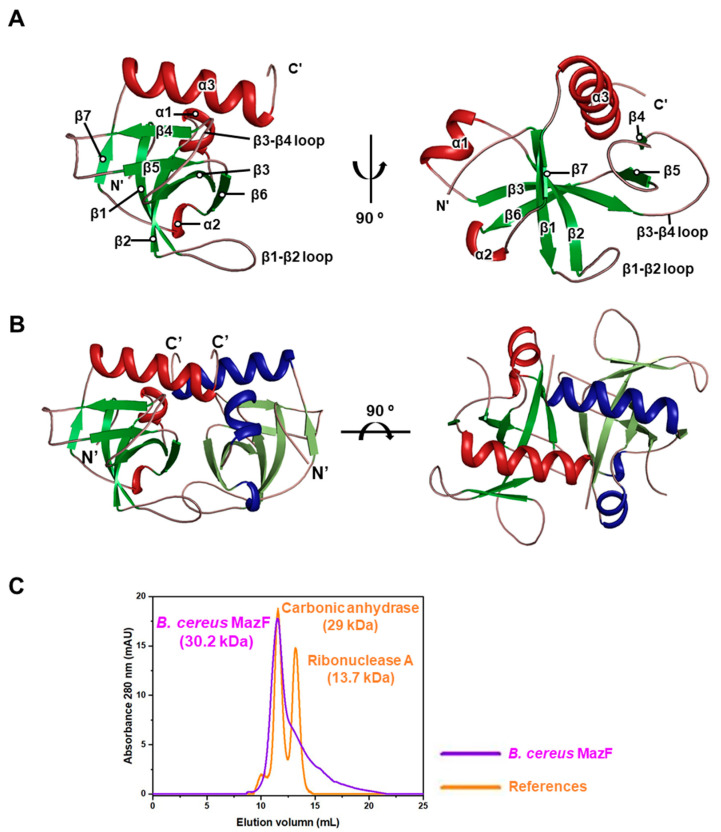
Overall structure of *B. cereus* MazF and gel filtration data with reference proteins. α helices are colored in red and blue. β strands are colored in green. (**A**) 90° rotational views on *B. cereus* MazF monomer. β1–β2 and β3–β4 loops are denoted. (**B**) 90° rotational views on *B. cereus* MazF homodimer. (**C**) Molecular weight estimates of *B. cereus* MazF obtained by size-exclusion chromatography with Superdex 75 10/300 gl column. Overlaid chromatograms were denoted with their names and molecular weights.

There are three α-helices and seven β-strands with the order of β-barrel arrangement in a monomer of *B. cereus* MazF (Figure 1A). Among those β-strands, five β-strands (β1, β2, β3, β6, β7) and two β-strands (β4, β5) form a β-sheet antiparallel to each other. Two loops between the β1 and β2 strands, and the β3 and β4 strands are also clearly observed. The total solvent-accessible surface area of the monomeric structure is 6740 Å^2^, and the contact area between two monomers is 1440 Å^2^ (21.4% per monomeric subunit). The crystal structure shows that the dimeric interface displays a concave structure covered by the neighboring loop (Figure 1B). Calculations of surface and interface areas were conducted using the PISA server [30]. The oligomeric state of *B. cereus* MazF was predicted through size-exclusion chromatography with reference proteins in the Gel Filtration Calibration Kits (GE Healthcare, Chicago, IL, USA) (Figure 1C). Because theoretical molecular weight of the *B. cereus* MazF monomer containing N-terminal His-tag is 15.1 kDa, it is reasonable that oligomeric state of *B. cereus* MazF is homodimer (30.2 kDa) as it was eluted at almost the same time as that of carbonic anhydrase (29 kDa).

### 2.2. Comparison of B. cereus MazF with MazF Homologs

**Figure 2:**
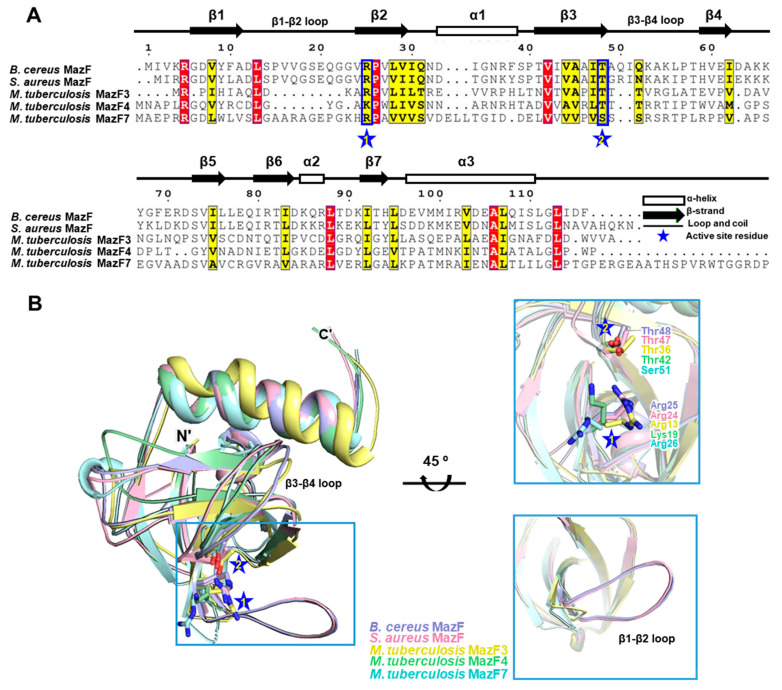
Comparative analysis of MazF toxins. (**A)** Sequence alignment of *B. cereus* MazF with other MazFs. Secondary structural elements are displayed above the alignment. Residues showing similarity are highlighted in red and yellow. Conserved active site residues are emphasized with star symbol. (**B**) Structural comparison of *B. cereus* MazF with other MazFs. Cartoon representations are employed to draw each structures. Conserved active site residues are shown in sticks. Conserved active site residues and variations in β1–β2 loop are illustrated by enlarged view in blue square.

To relate the structural characteristics of *B. cereus* MazF with mRNA interferase MazF, comparative analysis was performed with previously reported MazF structures. Illustrations of sequence alignment were displayed with *Staphylococcus aureus* MazF (PDB code 4MZM) [31], *M. tuberculosis* MazF3 (PDB code 5UCT), [32]; MazF4 (PDB code 5XE2) [29]; and MazF7 (PDB code 5WYG) [33] (Figure 2A). The results showed that two key residues of Arg25 and Thr48 involved in catalytic activity are well conserved among multiple MazFs. Interestingly, Lys19 is conserved in *M. tuberculosis* MazF4 instead of arginine [29], and Ser51 is conserved in *M. tuberculosis* MazF7 instead of threonine [33]. Yet, mutations of these two residues significantly impaired the activities of corresponding toxins, implying their role in ribonucleic catalysis [29,33]. Although considerable structural similarities are shared between the compared MazFs, there are notable differences in flexibilities and structural variations in their β1–β2 and β3–β4 loops (Figure 2B). These loops are reported as a major binding site with its cognate antitoxin MazE and, therefore, it is supposed that the flexibilities of these loops have relations to interactions with its target RNA substrate depending on MazE binding [32,34,35].

Through early studies on *E. coli* MazEF, it was suggested that the β1–β2 loop in the MazF toxin undergoes conformational transition from disordered to ordered state upon binding to the MazE antitoxin [36]. During this transition, it is assumed that these loops are modulated by MazE, affecting the target RNA accessibility of MazF [36]. For instance, MazF4 and MazF7 toxins from *M. tuberculosis* with truncated β1–β2 loops had significant steric clashes within the β1–β2 loop region due to the high flexibility arising from the structurally unbound MazE antitoxin [29,33]. As for MazF3 toxin in *M. tuberculosis*, the length of the short β1–β2 loop may be insufficient to reach and recognize the downstream of the target RNA substrate [31,32]. Furthermore, it is predicted that absence of an elongated β1–β2 loop would cause even greater conformational transition of the β1–β2 loop, leaving the toxin structurally and functionally impractical. Therefore, it is suggested that target sequence specificity of truncated or short β1–β2 loops would be significantly reduced compared with MazFs having a long β1–β2 loop, such as that of *B. cereus* MazF. Therefore, it can be presumed that the long length of β1–β2 loop may be the most powerful factor that determines the target RNA specificity.

In contrast to the β1–β2 loop, β3–β4 loop shows an opposite conformational transition from ordered to disordered state upon binding to MazE antitoxin [29]. Furthermore, previous research has shown that aromatic residues located on the antitoxin MazEs are involved in essential MazEF interactions [29]. Specifically, His68 of *E. coli* MazE, Tyr61 of *B. subtilis* MazE, and Tyr76 of *M. tuberculosis* MazE4 interacted with the β3–β4 loops via van der Walls interactions [29,34,35].

Thus, considering the *E. coli* and *M. tuberculosis* model proteins, the *B. cereus* MazE antitoxin, BC0265, likely contains C-terminal aromatic residues that might interact with the β3–β4 loop in *B. cereus* MazF. Because of high structural unpredictability and lack of structural information of *B. cereus* MazE and other MazE antitoxins in general, an accurate prediction of which aromatic residue will interact with its cognate toxin is difficult. However, assuming the model is correct, one of the aromatic residues in *B. cereus* MazE, Tyr63, Phe78, Tyr82 or His86, likely engages in a hydrophobic interaction during complex formation.

### 2.3. Arg25 and Thr48 Act as Key Residues in Catalytic Activity

**Figure 3:**
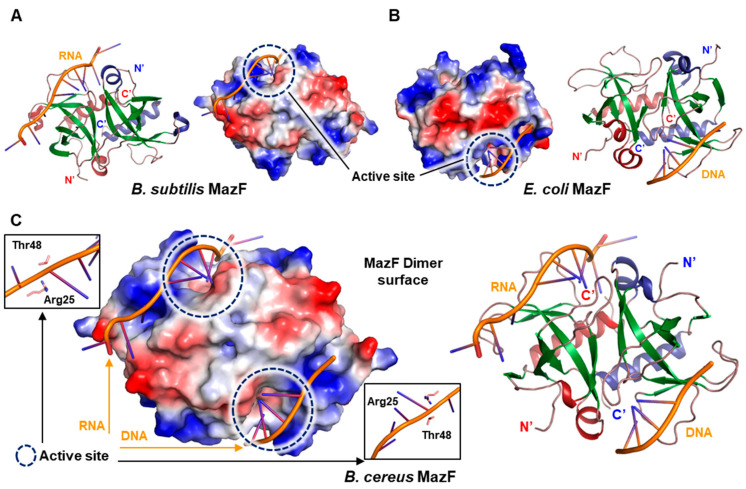
In silico model of previously reported MazFs and *B. cereus* MazF in complex with RNA and DNA. (**A**–**C**) Active sites for each proteins are illustrated in circles. (**A**) Ribbon representations and electrostatic surface potentials of *B. subtilis* MazF complexed with RNA. RNA is handled to be placed at left chain. (**B**) Ribbon representations and electrostatic surface potentials of *E. coli* MazF complexed with DNA. DNA is handled to be placed at right chain. (**C**) Superimposition of *B. cereus* MazF on *B. subtilis* MazF complexed with RNA (left chain) and *E. coli* MazF complexed with DNA (right chain). Close-up views of the interactions between active site residues and RNA/DNA are displayed.

To assure that two key residues Arg25 and Thr48 are critical in catalytic activity, comparative analysis with *B. subtilis* MazF complexed with RNA (PDB code 4MDX) [34] (Figure 3A) and *E. coli* MazF complexed with DNA substrate analog (PDB code 5CR2) [35] (Figure 3B) was performed. Subsequently, in silico model of *B. cereus* MazF complexed with RNA and DNA was generated by superimposition of *B. cereus* MazF structure onto the 4MDX (left) and 5CR2 (right), respectively (Figure 3C).

In the in silico model of *B. cereus* MazF in complex with RNA and DNA substrate, Arg25 and Thr48 residues were well organized with RNA and DNA substrate (Figure 3C). Moreover, mutational study on *B. subtilis* MazF Arg25 and Thr48 resulted in complete inactivation of toxicity of MazF [34]. From these results, it can be assumed that Arg25 and Thr48 from *B. cereus* would also be involved in RNA catalysis. In addition, according to earlier work performed on MazF toxins, Arg25 is predicted to act as a general acid/base during RNA catalysis and Thr48 may serve as a charge stabilizer in transition state during RNA substrate binding [35,37,38].

Interestingly, charge distributions observed in the front surfaces of three proteins (Figure 3A–C) are similar but slightly different from each other, mainly due to the differences in the charged amino acid content. In *E. coli* MazF, negative-charged front surface is mostly formed by Asp16, Asp18, and Glu24 in the β1–β2 loop (residues 16 to 27). However, corresponding residues are substituted with different amino acids in both *B. subtilis* and *B. cereus* MazF. Because these front surfaces are known as the binding region of cognate antitoxin MazE, each MazE is thought to have a surface potential specific to its cognate MazF toxin to effectively bind and neutralize toxin surfaces. This seems to be the one of strong reasons why a specific toxin is neutralized only by its cognate antitoxin, although considerable structural similarities are observed within the same family of TA system [29,39,40].

### 2.4. B. cereus MazF Shows Ribonuclease Activity by Two Key Residues

**Figure 4:**
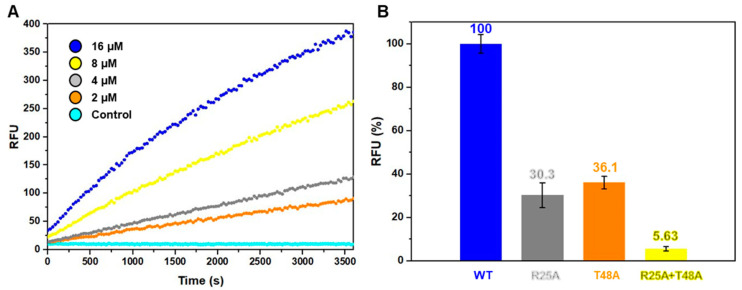
Ribonuclease activity test of *B. cereus* MazF toxin. (**A**) The in vitro ribonuclease activity test of wild-type *B. cereus* MazF. Fluorescence was measured as a function of time (s) during 1 h. Each curve is colored separately according to different *B. cereus* MazF concentrations. Concentrations of *B. cereus* MazF were increased by doubling from 2 to 16 μM. An equal aliquot of fluorescent RNA substrate was incubated with *B. cereus* MazF in different concentrations. Fluorescence was measured upon cleavage of RNA substrate. The control as well as different protein concentrations contained 20 mM Tris-HCl (pH 8.0), 500 mM NaCl, and 40 units of RiboLock™ (Thermo Scientific, Waltham, MA, USA) RNase inhibitor. (**B**) Ribonuclease activities of 10 μM wild-type *B. cereus* MazF and its mutants represented as bar graph with standard deviation derived from triplicate tests. Magnitudes of each reaction were assessed by subtracting the RFU (resulting fluorescence unit) of the starting point from RFU of the end point. The RFU obtained from wild-type *B. cereus* MazF was taken as 100%.

MazF is a ribonuclease that is known to cleave RNA substrate [41]. Toxins such as VapC in the Type II TA system require metal ions as a cofactor to exhibit ribonuclease activity, but in the case of MazF, cofactor metal is not necessarily needed [42,43]. To demonstrate that *B. cereus* MazF shows ribonuclease activity like other MazF toxins, in vitro ribonuclease activity test was performed (Figure 4A). In this assay, a synthetic RNA strand of unknown sequence is used as substrate. A fluorophore is covalently attached to one end of the RNA strand while the quencher is located at the other end. If this RNA is cleaved by a ribonuclease, the quencher is detached and as a result fluorescence is detected depending on the amount of cleaved RNA. The result from in vitro ribonuclease assay showed an elevation of RFU (resulting fluorescence unit) as a function of time. *B. cereus* MazF exhibits a ribonuclease activity showing time and dose dependency, and hence shares the same functional activity with MazF toxins.

Furthermore, two single and one double site-directed mutagenesis on the two key catalytic residues of Arg25 and Thr48 were performed (Figure 4B). The results of the mutational studies showed that single mutations of either Arg25 or Thr48 resulted in severe reductions in the ribonuclease activity of toxin while double mutations of both Arg25 and Thr48 resulted in close to none ribonuclease activity. Therefore, in agreement with the results of *B. subtilis* MazF, Arg25 and Thr48 are key residues in *B. cereus* MazF, which is a characteristic observed consistently in MazF toxins.

## 3. Conclusions

We conducted an in-depth characterization and investigation on putative mRNA interferase MazF in *B. cereus*. High-resolution (2.0 Å) structure was obtained by X-ray crystallography, and overall structural folding of *B. cereus* MazF indicated β-barrel arrangement containing two independent β-sheets identical to that of MazF toxins. Through comparison analysis with previously reported structures of MazFs, amino acid residues Arg25 and Thr48 in *B. cereus* MazF were well conserved with other MazFs and were also confirmed as key residues in the catalytic activity of *B. cereus* MazF. Also, β1–β2 and β3–β4 loops were clearly observed in *B. cereus* MazF structure and their role in the recognition of target RNA substrate depending on the binding of its cognate antitoxin was discussed. From previously conducted studies, it can be presumed that the β1–β2 loop acts as a gateway to binding of its target RNA substrate and cognate antitoxin and the β3–β4 loop also acts as a major interaction site in antitoxin-binding via van der Walls interactions. Lastly, ribonuclease activity test via in vitro assay together with site-directed mutational studies on Arg25 and Thr48 showed a decrease in its ribonuclease activity, which was consistent in other MazF toxins. Altogether, our study provides a unified structural and functional basis that mRNA interferase BC0266 is indeed a MazF toxin. This is the first determined MazF and toxin structure in type II TA system of *B. cereus.* Our work may be a rational basis for understanding the TA systems regarding the stress-responsive toxin MazF and the general regulatory mechanisms of TA systems.

## 4. Materials and Methods

### 4.1. Cloning and Transformation

The *BC0266* gene was amplified by polymerase chain reaction (PCR). The following primers were used in PCR: forward, 5′- GGAATTCCATATGATGATTGTAAAACGCGGC-3′; reverse, 5′- CCGCTCGAGTTAAAAATCTATTAGTCCTAAAC-3′. *Nde1* and *Xho1* were used as restriction enzymes, and the cutting sites are underlined. The PCR products and pET28a vector were double-cleaved by the same restriction enzymes and ligated to each other. For purification, residual N-terminal His tag (MGSSHHHHHHSSGLVPRGSH) was attached to the gene of *B. cereus* MazF and was transformed into *E.coli* DH5α competent cells (Novagen, Madison, WI, USA). As the next steps, these cloned plasmids were re-transformed into *E. coli* Rosetta2 (DE3) pLysS competent cells (Novagen, Madison, WI, USA).

### 4.2. Protein Expression and Purification

Transformed cells were grown in Luria broth (LB) at 37 °C until the OD_600_ reached to 0.5. Overexpression of target protein was induced by 0.5 mM isopropyl 1-thio-B-D-galactopyranoside (IPTG). Expressed cells were further incubated for 4 h at 37 °C. These cells were harvested by centrifugation at 11,355× *g*. Harvested cells were suspended in a buffer A (20 mM Tris-HCl, pH 7.9, and 500 mM NaCl) containing 5% glycerol and lysed by ultrasonication. Lysed cells were centrifugated for 1 h at 28,306× *g*. After centrifugation, the supernatant containing soluble moiety of protein was loaded on a Ni^2+^ affinity chromatography column (Bio-Rad, Hercules, CA, USA), which was pre-equilibrated with buffer A. Loaded protein was washed with buffer A containing 50 mM imidazole to remove impurities. Then, the remaining protein, bound to the Ni^2+^ column, was eluted using an imidazole gradient (100−800 mM) and fractionized. The purity of eluted protein in each fraction was checked using sodium dodecyl sulfate polyacrylamide gel electrophoresis (SDS−PAGE). Finally, selected high-purity protein was loaded on a size-exclusion chromatography column HiLoad 16/600 Superdex 200 prep-grade column (GE Healthcare, Chicago, IL, USA), pre-equilibrated with final buffer (20 mM Tris, pH 8.0, and 500 mM NaCl). Final eluted protein was concentrated to 20 mg/mL using an Amicon Ultra centrifugal filter unit (Millipore, Burlington, MA, USA). The purity of the final protein solution was verified by SDS-PAGE. Mutants of *B. cereus* MazF were expressed and purified by the same procedure as native ones.

### 4.3. Crystallization, Data Collection, and Processing

The final protein solution was screened for crystallization using crystallization kit Wizard (Rigaku Reagents, Bainbridge Island, WA, USA) and Index (Hampton Research, Aliso Viejo, CA, USA). Protein solution (1 μL) was mixed with 1 μL of each buffer solution in the crystallization kit. Crystals of *B. cereus* MazF were grown in the transparent 96-well plate using sitting-drop vapor diffusion method at 20 °C. Only one crystallized well contained the hit solution of 100 mM Sodium Citrate, pH 5.6, and 1.0 M ammonium phosphate monobasic. Cryo-protection was achieved by addition of 20% glycerol to the hit solution. After cryo-protection, crystals were immediately cooled in liquid nitrogen prior to data collection. The data were collected using an Rayonix MX-300 HE CCD detector at BL44XU of the Spring-8, Japan. All raw data of crystal were scaled and processed by XDS [44]. A set of data from a hit crystal was used to solve the structure at 2.00 Å resolution and to refine 7BXY (code name of *B. cereus* MazF). *PHENIX* [45] was first used to do molecular replacement and automatically build the model, and *COOT* [46] was utilized to yield the starting model for refinement. The *R_work_*/*R_free_* values [47] of the final model were obtained using *REFMAC* and *PHENIX* [45,48]. The validation of overall geometry was achieved using *MolProbity* [49]. PyMOL (PyMOL Molecular Graphics System, Version 1.2r3pre, Schrödinger, LLC, NY, USA) was used to generate figures in this study.

### 4.4. Mutiple Sequence Alignment

To conduct sequence alignment of *B. cereus* MazF and four MazFs whose structures have been identified, the sequence information of five proteins was browsed using Uniprot [50]. Alignments of amino acid residues were carried out using Clustal W [51] and visualized using ESPript 3.0 [52]. The consensus value was set to 0.85 and %Equivalent was used in similarity mode. In visualization, structural information of *B. cereus* MazF was used as top secondary structures and the description and sequence numbering on the topside correspond to *B. cereus* MazF.

### 4.5. In Vitro Ribonuclease Assay

To confirm the ribonuclease activity of *B. cereus* MazF, an RNase Alert Kit (IDT, Coralville, IA, USA) was purchased and used following the manufacturer’s protocol. Equal aliquot RNA substrate (5 μL) was interacted with 2 μM, 4 μM, 8 μM, and 16 μM concentrations of *B. cereus* MazF in a final purification buffer. For ribonuclease activity test, 10 μM aliquots of wild-type *B. cereus* MazF and its mutants were prepared. Then, the resulting fluorescence units (RFU) were detected using a SPECTRAmax GEMINI XS spectro-fluorometer (Marshall Scientific, Hampton, NH, USA) at 37 °C by emission fluorescence at 520 nm upon excitation at 490 nm. Assay setting was performed on 384-well opaque plate, and all of experiments were performed in triplicate.

### 4.6. Site-Directed Mutagenesis

The mutations in *B. cereus* MazF (R25A, T48A, R25A+T48A) were performed using *B. cereus* MazF in pET28a (+) as template. Single-site mutations were performed in one step, and double-site mutations were performed in a step-wise manner. Reaction components were mixed and subjected to PCR machine guided by the manufacturer’s protocol (EZchange Site-Directed Mutagenesis Kit, Enzynomics, Daejeon, Korea). Each plasmid was transformed into *E. coli* XL10-Gold competent cells (Agilent Technologies, Santa Clara, CA, USA), and the resulting inserted genes were verified through DNA sequencing.

## Figures and Tables

**Table 1:** Structure data collection and refinement statistics.

(a) Data Collection Details	
X-ray source	BL44XU beamline of Spring-8, Japan
X-ray wavelength (Å)	0.899995
Space group	*P3_1_21*
Unit cell parameters: *a*, *b*, *c* (Å)	60.648, 60.648, 76.247
Unit cell parameters: α, β, γ (°)	90.0, 90.0, 120.0
Resolution range (Å)	50.0-2.00
Observed reflections (>1σ)	231077
Unique reflections	21852
<I/σ(I)>	10.74 (2.97) ^e^
Completeness (%)	99.3 (95.5) ^e^
Multiplicity ^a^	10.57 (9.76) ^e^
*R*_merge_ (%) ^b^	12.1 (47.9) ^e^
CC_1/2_	0.997 (0.918) ^e^
**(b) Refinement statistics**	
*R*_work_^c^ (%)	18.9
*R*_free_^d^ (%)	22.5
No. of atoms/average *B* factor (Å^2^)	997/46.2
RMSD ^f^ from ideal geometry: Bond distance (Å)	0.008
RMSD ^f^ from ideal geometry: Bond angle (°)	1.108
Ramachandran statistics: Most favored regions (%)	96.49
Ramachandran statistics: Additional allowed regions (%)	3.51
PDB accession code	7BXY

^a^*N*_obs_/*N*_unique_, ^b^*R*_merge_ = Σ (*I* − 〈 *I* 〉)/Σ 〈 *I* 〉, ^c^ *R*_work_ = Σ*_hkl_* ||F_obs_| − *k* |F_calc_||/Σ*_hkl_* |F_obs_|, ^d^ *R*_free_ was calculated in the same manner as *R*_work_ with 5% of the reflections excluded from the refinement. ^e^ Values in parentheses indicate the highest-resolution shell. ^f^ Root mean square deviation (RMSD) was calculated using REFMAC.
